# The Landscape of Exosome-Derived Non-Coding RNA in Leukemia

**DOI:** 10.3389/fphar.2022.912303

**Published:** 2022-06-15

**Authors:** Bing-Jie Tang, Bao Sun, Lei Chen, Jie Xiao, Shu-Ting Huang, Ping Xu

**Affiliations:** ^1^ Department of Pharmacy, The Second Xiangya Hospital, Central South University, Changsha, China; ^2^ Institute of Clinical Pharmacy, Central South University, Changsha, China

**Keywords:** exosome, non-coding RNA, leukemia, tumor development, therapeutic target, biomarker

## Abstract

Leukemia is a group of life-threatening hematological malignancies which is currently incurable and often accompanied by drug resistance or disease relapse. Understanding the pathogenesis of leukemia and finding specific therapeutic targets and biomarkers is of great importance to improve the clinical efficacy of leukemia. Exosome-derived ncRNAs have been demonstrated as critical components of intercellular communication and function as key facilitators in the leukemia biological process. This review outlines the current investigations of exosomal ncRNAs (including miRNA, circRNA, and lncRNA) as important mediators of leukemia and potential therapeutic targets and biomarkers for leukemia treatment. Moreover, we generally analyze the prospects and challenges for exosomal ncRNAs from the aspects of research and clinical application.

## Introduction

Leukemia is a group of hematological malignancies characterized by the abnormal proliferation of hematopoietic cells that infiltrate the bone marrow and invade the blood and other extramedullary tissues (Bhat et al., 2020). Over 250,000 people worldwide are diagnosed with leukemia each year, accounting for 2.5% of all cancers (Rodriguez-Abreu et al., 2007). The four major subtypes of leukemia are acute lymphocytic leukemia (ALL), chronic lymphocytic leukemia (CLL), acute myeloid leukemia (AML), and chronic myeloid leukemia (CML), with different clinical features and prognoses (Rodriguez-Abreu et al., 2007) (Table 1). The fundamental form of treatment for leukemia is chemotherapy. But leukemia is incurable currently and patients often develop drug resistance or disease relapse (Mardani et al., 2019). In addition, the non-specific toxicity of traditional chemotherapeutic drugs to normal blood cells can cause serious hematological adverse reactions and affect the efficacy of the drugs. Therefore, demonstrating the pathogenesis of leukemia and finding specific therapeutic targets and biomarkers is of great importance to improve the clinical efficacy of leukemia patients.

**TABLE 1:** The characteristics of different types of leukemia.

	Pathophysiology	Epidemiology	Treatment	Prognosis
AML	Infiltration of the bone marrow, blood, and other tissues by proliferative, clonal, abnormally differentiated, and occasionally poorly differentiated cells of the hematopoietic system (Döhner et al., 2015).	Accounts for ∼30% of all leukemias in adults;	Continuous-infusion cytarabine with an anthracycline;	Adult patients <60 years old: cured in 35%–40%;
		The annual incidence rate in Europe ranges from two per 100,000/year to four per 100,000/year. (Rodriguez-Abreu et al., 2007)	Allogeneic Hematopoietic Stem Cell Transplantation (HSCT). (Döhner et al., 2015)	Adult patients >60 years old: cured in 5%–15%. (Döhner et al., 2015)
CML	CML is a clonal haemopoietic stem cell disorder characterised by a reciprocal translocation between the long arms of chromosomes 9 (ch9) and 22 (ch22), which produce the fusion BCR-ABL1 oncogene. (Apperley, 2015)	CML affects about one individual per 100,000 population per year with a slight male preponderance and accounts for 15% of all new cases of leukemia in the Western hemisphere. (Apperley, 2015)	TKIs: Imatinib, Nilotinib, Dasatinib;	Deep molecular responses were achieved in 69% and 56% of patients at 48 months (Geelen et al., 2017).
			Allogeneic HSCT (Apperley, 2015)	10%–15% of patients remain resistant to TKIs and at risk of disease progression (Apperley, 2015)
ALL	ALL is a malignant transformation and proliferation of lymphoid progenitor cells in the bone marrow, blood and extramedullary sites (Terwilliger and Abdul-Hay, 2017).	Represent ∼15% of leukemias in adults;	Most programs are centered on a vincristine, prednisone, and anthracycline combination	Long-term survival approaches 90% for standard-risk pediatric ALL (Terwilliger and Abdul-Hay, 2017);
		The most common form of leukemia in people <20 years old, accounting for over 80% of all leukemia patients and for 30% of all cancers in children. (Rodriguez-Abreu et al., 2007)	Allogeneic HSCT (Bassan and Hoelzer, 2011)	The average survival of patients age 18–60 years is 35% (Bassan and Hoelzer, 2011).
CLL	CLL is a malignancy of CD5^+^ B cells that is characterized by the accumulation of small, mature-appearing neoplastic lymphocytes in the blood, marrow and secondary lymphoid tissues, resulting in lymphocytosis, leukemia cell infiltration of the marrow, lymphadenopathy and splenomegaly (Kipps et al., 2017)	CLL is the most common leukemia in adults in many Western countries, but it is rare in Asia.	A combination of venetoclax with obinutuzumab, ibrutinib monotherapy, or chemoimmunotherapy.	Minimal residual disease (MRD)-positive patients: 10-years PFS is 10% and 10-years OS is 30%;
		CLL is estimated to account for ∼19,000 of all newly detected cancers in the United States in 2016. (Rodriguez-Abreu et al., 2007; Kipps et al., 2017)	Allogeneic HSCT (Hallek, 2019)	MRD-negative patients: 10-years PFS is 65% and 10-years OS is 70%. (Hallek, 2019)

**FIGURE 1:**
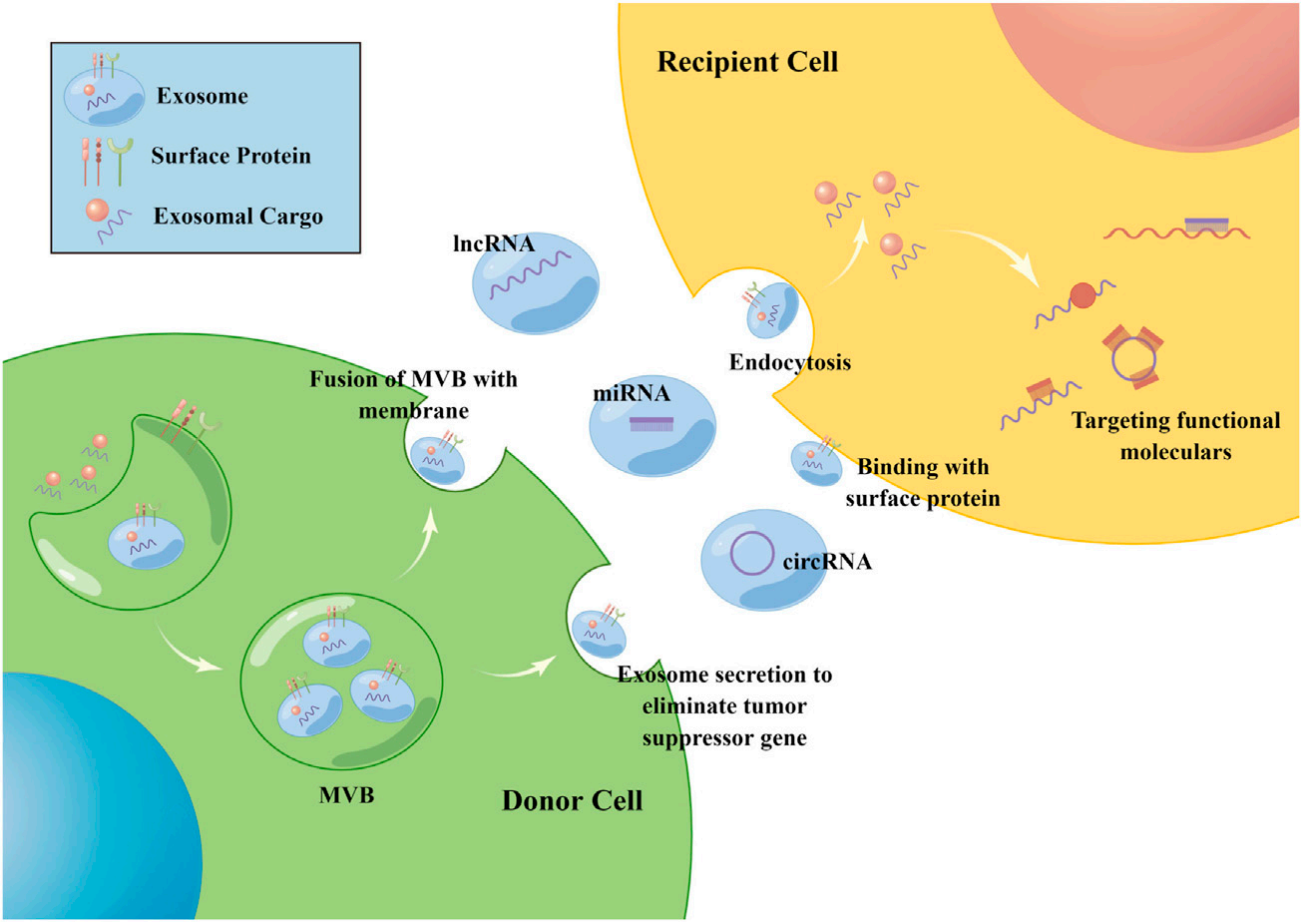
The biogenesis of exosomes. Exosomes are intraluminal vesicles of MVB and can be secreted from cells by fusion of MVBs with the plasma membrane, then internalized into recipient cells *via* endocytosis or the binding of surface proteins to release the encapsulated “cargo”. Non-coding RNAs including miRNA, circRNA and lncRNA can be encapsulated in exosomes and transmitted among cells. Once inside the recipient cells, ncRNAs can exert their function through targeting RNAs and proteins. The figure was drawn by Figdraw (www.figdraw.com).

**FIGURE 2:**
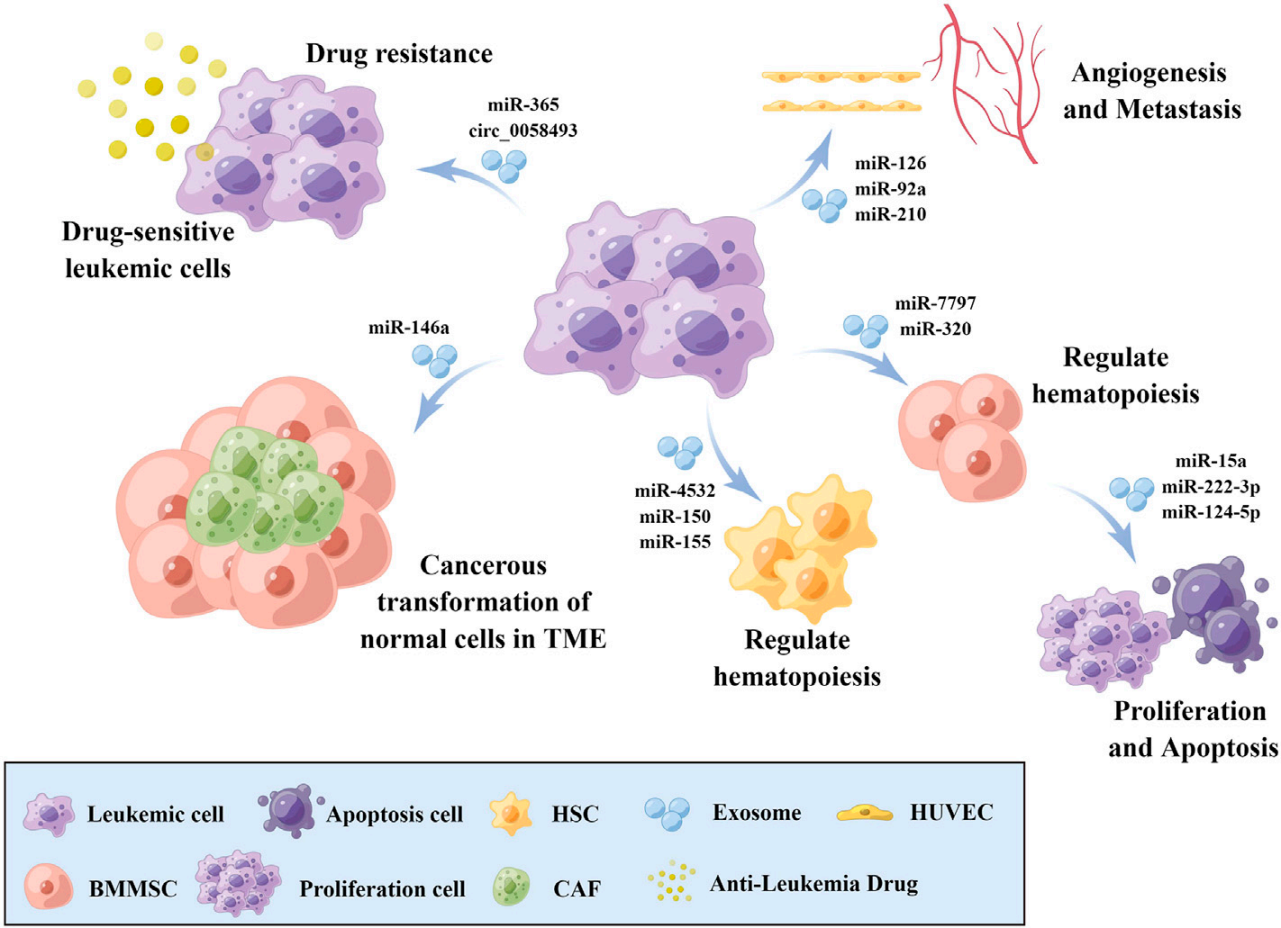
The role of exosomal ncRNAs in leukemia biology. Exosomal ncRNAs are involved in the cancerous transformation of stromal cells in the tumor microenvironment, drug resistance transmission, proliferation, apoptosis, angiogenesis, and metastasis *via* transmission among cells. BMMSCs: Bone marrow mesenchymal stem cell, HSCs: Hematopoietic stem cells, CAFs: Cancer-associated fibroblasts, HUVECs: Human umbilical vein endothelial cells, TME: Tumor microenvironment. The figure was drawn by Figdraw (www.figdraw.com).

Exosomes are intraluminal vesicles (ILV) of multivesicular bodies (MVB) and are typically 30–150 nm in diameter (Mashouri et al., 2019). A plethora of evidence indicated that exosomes could mediate tumor progression through participating in intercellular communication within the tumor microenvironment (TME) (Zhou et al., 2018; Mashouri et al., 2019). Exosomes can be secreted from cells by fusion of MVBs with the plasma membrane and internalized into recipient cells *via* endocytosis to release the encapsulated “cargo”, as well as regulated the biological processes (Figure 1). The composition of exosome cargo is heterogeneous and dynamic, which can reflect the unique profile of the constituents and more importantly, the physiological and pathological condition of their original cells or tissues. In addition to the proteins, nucleic acids, and lipids that have been detected in exosomes, non-coding RNAs (ncRNAs), a type of transcripts that are not translated into proteins, including microRNAs (miRNAs), long non-coding RNAs (lncRNAs), and circular RNAs (circRNAs), can also be encapsulated in exosomes and transmitted among cells. Among them, miRNAs are a type of small ncRNA of approximately 22 nucleotides in length with 5′-phosphate and 3′-hydroxyl ends (Slack and Chinnaiyan, 2019; Bhat et al., 2020). CircRNAs are single-stranded covalently closed circular transcripts without 5′ caps and 3′ tails (Qu et al., 2018). LncRNAs are linear transcripts with lengths exceeding 200 nucleotides (Bhat et al., 2020). It had been reported that ncRNAs encapsulated in exosomes were involved in various pathological processes of tumors, such as the cancerous transformation of stromal cells in the tumor microenvironment (Noh et al., 2020), drug resistance transmission among cells (Zhang Z. et al., 2020), proliferation, apoptosis, angiogenesis, and metastasis (Zheng et al., 2018; Xie et al., 2020) (Figure 2). These functions make exosomal ncRNAs become an important source for the discovery of new tumor therapeutic targets.

Exosomes have been found in almost all the body fluids, such as plasma, serum, urine, semen, saliva, bronchial fluid, cerebral spinal fluid (CSF), breast milk, amniotic fluid, synovial fluid, tears, lymph, bile, and gastric acid (Guo et al., 2017; Doyle and Wang, 2019; Mashouri et al., 2019). Its detection in different body fluids is also evidence of its stability in a variety of adverse environments (Zhou et al., 2018). More importantly, exosomes can protect ncRNAs from ribonuclease degradation in blood circulation (Zhang et al., 2019). Furthermore, as exosomal ncRNAs can be stably detected in plasma or serum, they are regarded as a type of non-invasive liquid biopsy that may be more acceptable in clinical application. Besides, it has been confirmed in many types of cancer that the concentration of exosomes is much higher in the blood of cancer patients compared with healthy individuals, which can improve the detection sensitivity (Smolarz and Widlak, 2021). These characteristics highlight the potential of exosomal ncRNAs as both biomarkers and treatment vectors in tumor therapy.

The transfer of exosomal ncRNAs between cells and subsequent functional effects have been extensively demonstrated in numerous solid tumor studies, but their functions in leukemia have not been well explored. This review outlines the current understanding of exosomal ncRNAs expression patterns in different types of leukemia, the mechanisms that contribute to leukemia carcinogenesis, and their potential role in the clinical application (Table 2). Revealing the regulatory patterns of exosomal ncRNAs in leukemia can lead to the identification of novel therapeutic targets and biomarkers, ultimately opening new prospects for treatment, diagnosis, and prognostication of leukemia.

**TABLE 2:** The role of exosome-derived ncRNAs in leukemia biology.

Exosomal ncRNA species	Exosomal ncRNAs	Leukemia type	Origin	Biological function	Potential role in future treatment	References
miRNA	miR-532	AML	AML plasma	A high level of exosomal miR-532 is associated with overall survival	Prognostic biomarker	Lin et al. (2020)
	miR-124-5p	AML	BMMSCs	Suppresses AML progression	Therapeutic target	Xu Y. C. et al. (2020)
	miR-222-3p	AML	BMMSCs	Suppresses AML progression	Therapeutic target	Zhang F. et al. (2020)
	miR-34a	AML	LSCs	Exosome-mediated miR-34a knockdown in LSCs promotes AML progression	Therapeutic target	Hu et al. (2020)
	miR-7797	AML	AML cells	Repress BMMSCs hematopoiesis and enhance AML promotion	Therapeutic target	(Horiguchi et al., 2016; Yoshida et al., 2019)
	miR-4532	AML	AML cells	Repress HSC hematopoiesis and enhance AML promotion	Therapeutic target	Zhao et al. (2019)
	miR-125b	AML	AML serum	A high level of exosomal miR-125b is associated with a higher risk of relapse and overall death in intermediate-risk AML	Prognostic biomarker	Jiang et al. (2018)
	miR-34c-5p	AML	LSCs	Exosome-mediated miR-34c-5p knockdown in LSCs promotes AML progression	Therapeutic target	Peng et al. (2018)
	miR-101-3p	AML	BMMSCs	Significantly overexpressed in MSCs-Exo of AML patients compared with healthy donors	Diagnostic biomarker	Barrera-Ramirez et al. (2017)
	miR-23b-5p, miR-339-3p, miR-425-5p	AML	BMMSCs	Significantly downregulated in MSCs-Exo of AML patients compared with healthy donors	Diagnostic biomarker	
	miR-150, miR-155	AML	AML cells	Inhibit the hematopoietic function of HSCs	Therapeutic target	Hornick et al. (2016)
	miR-711	CML	CML cells	Suppress the adhesive function of BMMSC	Therapeutic target	Jiang et al. (2020)
	miR-15a	CML	hBMMSCs	Inhibit the Proliferation of CML Cells	Therapeutic target	Zhang X. et al. (2020)
	miR-320	CML	CML cells	Inhibit osteogenesis of BMMSCs and promote CML cells proliferation.	Therapeutic target	Gao et al. (2019)
			CML plasma	Significantly overexpressed in plasma exosomes of CML-BC patients compared with CML-CP patients	Prognostic biomarker	
	miR-92a-3p	CML	CML cells	Inhibit adipogenesis and promote CAC	Therapeutic target	Wan et al. (2019)
	miR-365	CML	CML cells	Mediate the transfer of imatinib-resistance	Therapeutic target	Min et al. (2018)
	miR-215	CML	CML plasma	Downregulated exosomal miR-215 indicates successful discontinuation of imatinib	Prognostic biomarker	Ohyashiki et al. (2016)
	miR-21	CML	CML cells	Mediate the anticancer effect of Curcumin in CML	Therapeutic target	(Taverna et al., 2015; Taverna et al., 2016)
	miR-126	CML	CML cells	Inhibit motility and adhesion of CML cells and promote CML progression.	Therapeutic target	Taverna et al. (2014)
	miR-92a	CML	CML cells	Enhance cell migration and tube formation.	Therapeutic target	Umezu et al. (2013)
	miR-210	CML	Hypoxic leukemia cells	Enhance tube formation in endothelial cells	Therapeutic target	Tadokoro et al. (2013)
	miR-146a	CLL	CLL cells	Induces the transition of MSCs to CAFs	Therapeutic target	Yang et al. (2020)
	miR-155	CLL	CLL cells	Mediate MDSCs induction and CLL immune defects	Therapeutic target	Bruns et al. (2017)
	miR-202-3p	CLL	CLL cells	Promote CLL progression	Therapeutic target	Farahani et al. (2015)
			CLL plasma	Significantly overexpressed in plasma exosomes of AML patients compared with healthy donors	Diagnostic biomarker	
	miR-150, miR-146a	CLL	CLL cells	miR-150 and miR-146a were enriched in CLL exosomes and could be transferred to BMMSCs	Therapeutic target	Paggetti et al. (2015)
	miR-29a-c, miR-150, miR-155	CLL	CLL plasma	Significantly overexpressed in plasma exosomes of CLL patients compared with healthy donors	Diagnostic biomarker	Yeh et al. (2015)
	miR-181a	ALL	ALL cells	Enhance ALL cells proliferation and Contribute to ALL chemoresistance	Therapeutic target	(Haque and Vaiselbuh, 2020a; b)
circRNA	circ_0004136	AML	AML cells	Exosome-mediated circ_0004136 knockdown restrains AML cell malignant progression through regulating miR-570-3p/TSPAN3 axis	Therapeutic target	Bi et al. (2021)
	circ_0009910	AML	AML cells	Enhance AML progression through regulating miR-5195-3p/GRB10 axis	Therapeutic target	Wang et al. (2021)
	circ_0058493	CML	CML cells	A high level of exosomal circ_0058493 is associated with IM resistance	Prognostic biomarker	Zhong et al. (2021)
	circRNA mc-COX2	CLL	CLL plasma	A high level of exosomal circRNA mc-COX2 is associated with CLL progression and prognosis	Prognostic biomarker	Wu et al. (2020)

## Role of Exosomal miRNAs in Leukemia

The most extensively studied class of ncRNA in exosomes to date is miRNA. MicroRNAs are short ncRNAs of approximately 22 nucleotides in length that regulate the expression of other RNAs, notably mRNAs, through binding between the 5’ end of the miRNA with complementary sequences in target RNAs (Slack and Chinnaiyan, 2019). Exosomal miRNAs have been implicated in a host of the biological process of cancer cells (Nicoloso et al., 2009) and have been reported to be involved in tumor cell proliferation, apoptosis, invasion, migration, angiogenesis, the transformation of the tumor microenvironment, and the occurrence and transfer of drug resistance. Exosomal miRNAs are expected to become a new type of therapeutic target and biomarker.

### Role of Exosomal miRNAs in Acute Myeloid Leukemia

Acute myeloid leukemia (AML) is the most common acute leukemia in adults, with a 5-year survival rate of 24% (Zhang F. et al., 2020). 30% of newly diagnosed patients cannot achieve complete remission, and about 50% of patients relapse after intensive chemotherapy (Thol et al., 2015). The main treatments for AML are allogeneic stem cell transplantation and intensive chemotherapy at present. However, the prognosis of AML patients, especially elderly patients, is poor (Dombret and Gardin, 2016; Shallis et al., 2019). Therefore, it is urgently needed to identify effective and reliable biomarkers and therapeutic targets for improving the prognosis of AML (Fang et al., 2020). Additionally, as the diagnosis of AML is often demonstrated by an increased number of myeloblasts in the bone marrow using marrow aspirate, the utilization of non-invasive tools for the diagnosis and management of AML will greatly reduce patients’ pain and the worldwide health burden (Fayyad-Kazan et al., 2013).

Bone marrow mesenchymal stem cell (BMMSC) plays a crucial role in regulating bone marrow hematopoietic function under physiological conditions and is considered to be a keystone of the hematopoietic stem cell niche (Zhang F. et al., 2020). Hematopoietic insufficiency is a hallmark of AML and MSCs from AML patients exhibit significant growth deficiency and impaired osteogenic differentiation capacity (Zhang F. et al., 2020). Yoshida et al. (2019) found that miR-7797 was significantly overexpressed in exosomes produced by AML cells, and could be transmitted into BMMSCs through exosomes, thereby regulating the Hippo-YAP signaling pathway, inhibiting the normal hematopoietic function of BMMSCs and promoting the development of AML (Horiguchi et al., 2016). Conversely, BMMSCs can also deliver ncRNAs through exosomes to regulate AML cell function. Zhang F. et al. (2020) found that miR-222-3p was significantly enriched in human BMMSCs exosomes which could be transmitted into AML cells, regulated the IRF2/INPP4B signaling pathway, inhibited the proliferation, and promoted the apoptosis of AML cells. Xu Y. C. et al. (2020) found that miR-124-5p mediated the inhibitory effect of BMMSCs exosomes on the cell cycle progression, AML cells proliferation, and apoptosis promotion. These findings indicate that exploring the BMMSCs-associated process may lead to the discovery of new therapeutic targets for AML.

AML is a genetically heterogeneous clonal disorder characterized by the accumulation of acquired genetic alterations in hematopoietic progenitor cells that alter normal mechanisms of self-renewal, proliferation, and differentiation, all of which are implicated in the process of hematopoiesis (Marcucci et al., 2011). Zhao et al. (2019) found that miR-4532 was significantly overexpressed in AML cell lines compared with CD34^+^ hematopoietic stem cells (HSCs) and enriched in AML cell exosomes. Exosomal miR-4532 could be transferred into HSCs and repress normal HSC hematopoiesis *via* activation of the LDOC1-dependent STAT3 signaling pathway. Hornick et al. (2016) found that exosomes derived from AML cells could destroy hematopoietic function through transferring miR-150 and miR-155 to hematopoietic stem cells and promote AML development. These findings indicate that AML cells can disturb normal hematopoietic function through exosome-derived miRNAs.

Emerging evidence has proved that AML originates from a small subpopulation of leukemic stem cells (LSCs) (Lapidot et al., 1994; Bonnet and Dick, 1997; Blair et al., 1998). LSCs are more resistant to conventional chemotherapy than ordinary leukemia cells and have the potential of self-renewal and initiating leukemia (Bonnet and Dick, 1997). Therefore, finding strategies to specifically eliminate LSCs may be necessary for solving AML recurrence or drug resistance (Peng et al., 2018). Hu et al. (2020) found that miR-34a was elevated in exosomes derived from LSCs. miR-34a could impede the secretion of exosomes which was promoted by RAB27B through inhibiting the expression of RAB27B. This process could increase the miR-34a level of LSCs in which miR-34a could inhibit HDAC2 expression and regulated the JAK1/STAT2/p53 pathway to promote the clearance of LSCs. However, LSCs took away the tumor-suppressive miR-34a by secreting exosomes, leading to the decrease of miR-34a in LSCs and the abnormal proliferation of LSCs (Hu et al., 2020). Similarly, miR-34c-5p has also been observed to promote LSCs clearance whereas RAB27B-mediated extracellular transfer of miR-34c-5p through exosomes partly promoted the miR-34c-5p decrease in LSCs, thus promoting the growth of LSCs (Peng et al., 2018). These studies suggest that inhibiting the extracellular transfer of tumor-suppressive miRNAs mediated by exosomes may be utilized to improve clinical response in future AML treatment.

Exosomal miRNAs also have a strong potential for clinical application as novel biomarkers for leukemia treatment. Upregulation of miR-125b in plasma exosomes was reported by Jiang and colleagues and was an independent prognostic factor for patients with intermediate-risk AML that indicated a higher risk of relapse and overall death (*p* < 0.001 for both) (Jiang et al., 2018). Lin et al. (2020) found that a high level of miR-532 in plasma exosomes of AML patients was positively correlated with overall survival (OS) (*p* < 0.05). Through screening and verification, Barrera-Ramirez *et al.* described upregulation of miR-101-3p and downregulation of miR-23b-5p, miR-339-3p, and miR-425-5p (*p* < 0.05) in MSCs exosomes of AML patients, compared to healthy donors (Barrera-Ramirez et al., 2017). The dysregulation of exosomal miRNAs and their association with AML prognosis demonstrate their potential as convenient, noninvasive biomarkers for future therapies.

### Role of Exosomal miRNAs in Chronic Myeloid Leukemia

Chronic myeloid leukemia (CML) is a clonal hemopoietic stem cell disorder characterized by a reciprocal translocation between the long arms of chromosomes 9 (ch9) and 22 (ch22) (Apperley, 2015). CML affects about 1-2/100,000 population per year, and accounts for 15% of all new cases of leukemia (Apperley, 2015). As the first-line drugs for CML treatment, tyrosine kinase inhibitors (TKIs) such as imatinib, dasatinib, and nilotinib have significantly improved the survival rate of CML patients. However, real-world research showed that within the first 3 years after diagnosis, 44% of CML patients on first-line treatment discontinued their first-line treatment, of which 21% were due to TKI intolerance and 19% were due to treatment failure (Geelen et al., 2017). Resistance to TKIs is one of the main causes of poor efficacy in CML patients, and the mechanism is still unclear.

Reciprocal interactions between leukemic cells and BMMSC remodel the normal niche into a malignant niche, leading to leukemia progression (Gao et al., 2019). The study of Gao and colleagues demonstrated that HNRNPA1 (a type of RNA-binding proteins) -mediated exosomal transfer of miR-320 from leukemia cells to BMMSC is an important process of leukemia progression and could be a potential therapeutic target for CML. As a tumor-suppressive miRNA, miR-320 was found to be upregulated in exosomes and secreted by leukemic cells, resulting in increased proliferation of the donor cells. Interestingly, the secreted exosomes were significantly endocytosed by adjacent BMMSC and inhibited osteogenesis partially *via* β-catenin inhibition, further promoting tumorigenesis. Besides, blast crisis CML (CML-BC) patients had a much higher miR-320 expression in plasma exosomes, compared to chronic phase CML (CML-CP) patients, indicating its clinical relevance (Gao et al., 2019). The study of Jiang et al. (2020) revealed the elevation of miR-711 in exosomes derived from K562 cells compared to parental cells exosomes. K562 cell-derived exosomal miR-711 could be transferred into BMMSCs and weaken the adhesive abilities (Jiang et al., 2020). miR-15a was shuttled by human BMMSC-exosomes which might be associated with the proliferation of CML cells (Zhang X. et al., 2020).

It has been reported that the interaction between leukemic cells and endothelial cells is associated with leukemia progression. Taverna et al. (2014) found that miR-126 was overexpressed in LAMA84 exosomes compared to parental cells and shuttled into human umbilical vein endothelial cells (HUVECs). miR-126 could change the bone marrow microenvironment *via* inhibiting the expression of chemokine CXCL12 and adhesion molecule VCAM1 in HUVECs, resulting in decreased motility and adhesion of LAMA84 cells, potentially facilitating disease progression (Taverna et al., 2014). The study of Umezu et al. (2013) showed that exosomes derived from K562 cells could transfer miR-92a into HUVECs and promote proliferation and angiogenesis. Tadokoro et al. (2013) found K562 exosomes could transfer miR-210 into HUVECs and promote angiogenesis. These data indicate the intercellular communication among exosomal miRNAs and endothelial cells demonstrates a possibility as a future therapeutic target due to its effect on the tumor microenvironment.

Exosomal miRNAs have also been implicated in the drug resistance of CML. The study of Min et al. (2018) demonstrated that miR-365 was significantly upregulated in exosomes derived from CML IM-resistant cells compared to those from sensitive cells. The subsequent results demonstrated that IM-resistant cells could transfer exosomal miR-365 into sensitive cells and inhibited the expression of BAX and Cleaved caspase-3, inducing drug resistance in sensitive cells (Min et al., 2018). This suggests CML cells may utilize exosome-derived miRNAs to improve chemoresistance.

The use of TKIs makes the life span of CML patients close to healthy people, but lifelong medication may cause unacceptable side effects such as bone marrow suppression or great economic burden. Therefore, TKIs discontinuation has become a research hotspot of CML in recent years. The study of Ohyashiki et al. (2016) demonstrated exosomal miR-215 was downregulated in CML patients who successfully discontinued IM, indicating the obvious potential of miR-215 in plasma exosomes as a prognostic marker for IM discontinuation.

In addition, cancer-associated cachexia (CAC) has a significant influence on the treatment tolerance and life quality, thus affecting the treatment response of CML patients. miR-92a-3p was found highly expressed in CML-derived exosomes which could be taken up by adipose tissue and suppressed the adipogenic ability of adipose-derived mesenchymal stem cells (ADSCs), resulting in reduced body fat and weight in mice (Wan et al., 2019).

### Role of Exosomal miRNAs in Chronic Lymphocytic Leukemia

Chronic lymphocytic leukemia (CLL) is a malignancy of CD5^+^ B cells and characterized by the accumulation of small, mature-appearing lymphocytes in the bone marrow, blood, and lymphoid tissues (Kipps et al., 2017). CLL is the most common type of leukemia in western countries with a highly variable clinical course and is currently incurable (Dubovsky et al., 2013; Hallek, 2019). It is urgent to clarify the pathogenesis of CLL and find effective therapeutic targets for CLL.

Cancer-associated fibroblasts (CAFs) are one of the most crucial components of the tumor microenvironment and may derive from fibroblasts, epithelial cells, and BMMSCs (Xing et al., 2010). The intercellular transmission of miRNA through exosomes is an important factor in CAFs formation. Paggetti and colleagues found that exosomes derived from CLL cells could transfer miR-150 and miR-146a into BMMSCs. They also found CLL cell exosomes could induce the transformation of BMMSCs into CAFs, but whether it was mediated by miR-150 and miR-146a was not clearly stated (Paggetti et al., 2015). Subsequently, the study of Yang *et al.* demonstrated CLL cells exosomes could transmit miR-146a into BMMSCs and promote the CAFs transformation by inhibiting the expression of tumor suppressor protein USP16 (Yang et al., 2020). Their involvement in CAFs transformation hints at the possibility of using exosomal miRNAs as future targets for CLL treatment.

The intercellular communication among nurturing cells and leukemia cells through exosome transmission is also an essential factor for the formation of the tumor microenvironment. Farahani et al. (2015) found that miR-202-3p was enriched in CLL exosomes and could be transmitted into human stromal cells (HS-5), altering the transcriptome in the recipient cells and enhancing their proliferation. More importantly, the expression of miR-202-3p in exosomes derived from CLL patients’ plasma showed a higher level compared to healthy donors, indicating the potential of plasma exosomal miR-202-3p as a diagnostic biomarker in CLL treatment (Farahani et al., 2015).

CLL is characterized by immune defects that prevent an efficient anti-tumor response. The increase of myeloid-derived suppressor cells (MDSCs) is an important factor of CLL immunodeficiency. The study of Bruns et al. (2017) revealed that the transmission of miR-155 through CLL exosomes could induce the increase of MDSCs and this immune regulatory interplay could be disrupted by vitamin D that negatively regulated miR-155 expression in CLL-cells. More importantly, the microRNA profiling of plasma-derived exosomes conducted earlier by Yeh et al. (2015) identified distinct exosome microRNA signatures, including miR-29a-c, miR-150, and miR-155 that were significantly higher in circulating CLL exosomes compared to exosomes from healthy donors (*p* < 0.001). Besides, a high level of miR-150 and miR-155 in exosomes were strongly associated with BCR activation, the pathway which was essential in CLL development (Yeh et al., 2015). These data demonstrate the strong potential and advantages of exosomal miRNAs as convenient, noninvasive biomarkers and therapeutic targets for future leukemia treatment due to their easily accessible in peripheral blood and the extensive effect on tumor biological process.

### Role of Exosomal miRNAs in Acute Lymphocytic Leukemia

The estimated annual incidence of adult acute lymphocytic leukemia (ALL) is about one in 100,000. Most ALL occurs in children and adolescents younger than 20 years old, with cure rates approaching 90%. However, on the contrary, the therapeutic progress has been slow, with an average survival of 35% in adult patients aged 18–60 years (Bassan and Hoelzer, 2011; Man et al., 2017). Besides, relapsed leukemia is normally associated with high rates of treatment failure due to chemotherapy resistance (Haque and Vaiselbuh, 2020b). Exploring the relationships between ALL biological processes and exosome-associated non-coding RNAs can not only better clarify the pathogenesis of ALL but also provide novel therapeutic targets and biomarkers to improve ALL response. The study of Haque et al. revealed that exosomes derived from ALL cell lines promoted cellular proliferation not only in leukemia B cell lines but also in controlling human B cells. Furthermore, miR-181a was overexpressed in ALL cell exosomes and specific silencing of exosomal miR-181a reversed exosome-induced leukemia cell proliferation *in vitro*. These data suggested that exosomal miR-181a is a biologically active player with a functional role in leukemia cells and could be a novel target for growth suppression in ALL (Haque and Vaiselbuh, 2020a). Haque and colleagues further investigated the influence of vincristine and prednisone (the standard chemotherapeutic agents for ALL) on miR-181a expression in the first time diagnosed leukemia and relapsed leukemia. The consequences showed that vincristine and prednisone exposure did not change miR-181a expression either at the cellular or exosomal level in relapsed leukemia cell lines contrary to the first time diagnosed leukemia cells, where miR-181a expression was suppressed. More importantly, this non-suppressive nature of miR-181a made relapsed leukemia cells resistant to vincristine and prednisone. These data suggested that cellular and exosomal miR-181a played important roles in the chemoresistance of relapsed leukemia (Haque and Vaiselbuh, 2020b).

## Role of Exosomal circRNAs in Leukemia

Circular RNAs (circRNAs) are a class of single-stranded closed RNA molecules without 5′ caps and 3′ tails that undergo a specific backsplicing from pre-mRNA. circRNAs are found to be widely expressed across eucaryons and expressed specifically in cells and tissues (Qu et al., 2018). CircRNAs can competitively inhibit the function of miRNAs, acting as an RNA sponge to block miRNAs from binding to their target mRNA, regulating the expression of the relevant protein, and playing essential roles in the different biological processes of numerous tumors (Zhong et al., 2018).

Formed by backsplicing events where a 5′ cap binds to a 3′ tail, circRNAs lack 5′ and 3′ terminal ends. As such, circRNAs are inherently resistant to the ribonuclease of degradation, which works by targeting the 5′ and 3′ termini. This results in the high stability of circRNAs in the cytoplasm, exosomes, and body fluid. In fact, plenty of circRNAs has been detected in the peripheral circulation of patients and tumor-derived exosomes (Zhou et al., 2018). Moreover, Tay et al. (2015) investigated the design and effect of miRNA sponge expression vectors and found that circularized miRNA sponges were resistant to miRNA-mediated RNA destabilization and displayed superior anti-tumor activities compared to the linear carriers in malignant melanoma cell lines. The unique stability of circRNA endows it with a more potent inhibitory effect on the oncogenic activity of miRNAs and the advantages as potential therapeutic targets and biomarkers for future clinical applications.

To date, the role of exosomal circRNAs in leukemia has not been explored to the same extent as miRNAs. Nevertheless, there have been several recent studies that have begun to investigate their function in leukemia. The research of Wang et al. (2021) revealed that circ_0009910 could be shuttled among AML cells *via* exosomes and regulated the expression of miR-5195-3p and GRB10, thus influencing the proliferation, apoptosis, and cell cycle progression of AML cells. Bi et al. (2021) found that oncogene circ_0004136 could promote AML development through regulating miR-570-3p/TSPAN3 axis while exosome-mediated circ_0004136 knockdown restrained AML cell malignant progression. Our recent study also demonstrated that upregulated circ_0058493 in exosomes derived from CML cells was associated with imatinib resistance (Zhong et al., 2021). Besides, Wu et al. (2020) identified a novel mitochondrial genome-derived circRNAs (Mc), mc-COX2. They further found that elevated mc-COX2 in exosomes from CLL plasma compared to normal controls was associated with the progression and prognosis of CLL.

Currently, the understanding of the role of exosomal circRNA in the molecular mechanisms and possible clinical applications in leukemia are very limited and need further study. It has been extensively verified in solid tumors that exosomal circRNAs participate in numerous cancer biological processes including tumorigenesis, proliferation, angiogenesis, metastasis, drug resistance, etc (Liu et al., 2020; Xie et al., 2020; Xu J. et al., 2020; Huang et al., 2021). Xie et al. (2020) reported that exosomal circSHKBP1 regulated the miR-582-3p/HUR/VEGF pathway, suppressed HSP90 degradation, and promoted GC progression. Besides, circSHKBP1 derived from serum exosomes was more abundant in GC patients than in healthy controls, making it a promising circulating biomarker of GC diagnosis and an exceptional candidate for further therapeutic exploration. Xu J. et al. (2020) found that exosomes were crucial for spreading circRNA-SORE-mediated sorafenib resistance among HCC tumor cells. In addition, HCC patients with relatively lower exosomal circRNA-SORE expression had a higher response rate towards sorafenib (80%) compared with patients with higher expression (25%), indicating exosomal circRNA-SORE was a promising prognostic biomarker or therapeutic target for HCC treatment (Xu J. et al., 2020). These studies suggest that exosomal circRNAs have a powerful role in the regulation of the tumor biological process and further investigation is urgently needed to elucidate the precise function of exosomal circRNAs in leukemia.

### Role of Exosomal lncRNAs in Leukemia

LncRNAs are defined as transcripts with lengths exceeding 200 nucleotides that are not translated into protein and most of them are markedly expressed in differentiated tissues or particular cancer types (Bhat et al., 2020). Compared to small ncRNAs, lncRNAs exhibit extensive mechanistic diversity to carry out their function. Briefly, lncRNAs can exert their function through binding to protein complexes, nucleic acids and acting as ceRNAs that inhibit the effect of miRNAs, similarly to circRNAs (Slack and Chinnaiyan, 2019).

LncRNAs are involved in many crucial biological processes such as chromatin modification, gene expression, and nuclear transport, and are functional regulators of carcinogenesis, apoptosis, tumor migration, and drug resistance. Its functional relevance in cancer indicates the possibility of using lncRNAs as future biomarkers and therapeutic targets for cancer treatments (Zhou et al., 2018).

The function of exosomal lncRNAs in leukemia progression has not been reported yet, but recent studies have demonstrated that exosomal lncRNAs play critical roles in other hematological tumors. Li et al. (2018) found that lncRNA RUNX2-AS1 in myeloma cells could be packed into exosomes and transmitted to MSCs. RUNX2-AS1 was able to form an RNA duplex with RUNX2 pre-mRNA and transcriptionally repressed RUNX2 expression, resulting in decreased osteogenic potential of MSCs. Therefore, exosomal lncRNA RUNX2-AS1 may serve as a potential therapeutic target for bone lesions in MM (Li et al., 2018). The study of Deng et al. revealed that LINC00461 was highly expressed in MSC-derived exosomes and further enhanced multiple myeloma (MM) cell proliferation, which might become a candidate for therapeutic applications (Deng et al., 2019). Further investigations of the regulatory function and the clinical relevance of exosomal lncRNAs in leukemia are needed.

The function of exosome-derived lncRNAs has been investigated in a wide range of solid tumors and the results showed that exosomal lncRNAs could affect tumor growth, metastasis, invasion, and prognosis by regulating the tumor microenvironment. By intercellular transfer through exosomes, lncRNAs can create a microenvironment suitable for the metastasis of tumor cells (Zhou et al., 2018). Wu et al. (2018) found that exosomal lnc-MMP2-2 was involved in increasing vascular endothelial cell permeability and enhancing the invasion of lung cancer cells by promoting MMP2 expression. Their findings suggested that exosomal lnc-MMP2-2 might represent a putative therapeutic target for lung cancer treatment. The study of Behera and colleagues demonstrated both *in vitro* and vivo that lnc-H19 in BMMSC-exosomes significantly promoted endothelial angiogenesis and osteogenesis through binding with miR-106 and activating Angpt1/Tie2-NO signaling pathway in mesenchymal and endothelial cells (Behera et al., 2021). These findings demonstrated the mediating ability of exosomal lncRNAs in the pathological process of tumors. The emerging field of exosomal lncRNAs in leukemia is worth further exploration.

### Prospects and Challenges for Exosomal ncRNAs

Intercellular communication of cells *via* exosome-derived ncRNAs and subsequent functional effects have been demonstrated in numerous studies, including hematologic tumors and solid tumors, highlighting the potential of exosome-derived ncRNAs as functional mediators in tumors. On the one hand, exosomes derived from normal cells can transfer tumor suppressor genes into tumor cells, thus inhibiting tumor promotion. On the other hand, oncogenes can be shuttled from tumor cells into normal cells or sensitive cells, facilitating the formation of tumor microenvironment or drug resistance. Besides, tumor cells can reduce the cellular level of tumor suppressor genes through exosomes excretion, thus contributing to tumor promotion.

The regulatory function in the leukemia pathological process hints at the possibility of using exosomal ncRNAs as future therapeutic targets for leukemia treatments. According to how exosomal ncRNAs participate in tumor promotion, there are two research directions for future clinical application: 1) Increasing the level of tumor suppressor genes in a cancerous environment. a) Exosomes containing tumor suppressor genes with higher affinity for tumor tissues may be designed and synthesized through special membrane protein decoration. Then the functional exosomes are supposed to target the specific tumor microenvironment, increasing the level of tumor suppressor genes and inhibiting leukemia promotion. b) According to the research listed above (Table 2), functional ncRNAs are always enriched in exosomes and this phenomenon is controlled by certain loading and sorting mechanisms. Future studies should aim to regulate the loading and sorting process of exosomal ncRNAs and inhibit the secretion of tumor suppressor genes from tumor cells. 2) Decreasing the level of oncogenes in a cancerous environment. a) The protection of exosomes from enzymes in circulation makes it possible for the anti-RNAs loading in exosomes and being applied in gene therapy. In fact, Shtam et al. (2013) has transfected siRNA into exosomes and successfully silenced the expression of RAD51 using exosomes as vectors. b) As exosomes are critical components of cellular communication and the key facilitators in the exchange of information between cells (Zhou et al., 2018), it may be practical to inhibit the transmission of tumor-related exosomes in circulation or tumor microenvironment using target drugs or inhibitors.

To date, there have been abundant exosomal ncRNAs found to be dysregulated in leukemia and they are recognized as vital sources of diagnostic or prognostic biomarkers for future treatment. Exosomal ncRNAs are ideal biomarkers because of their: 1) Specificity: Specific types and concentration of ncRNAs in exosomes can represent the conditions of original cells or tissues; 2) Sensitivity: Emerging evidence have clarified that cancer patients have a larger number of circulating exosomes compared to healthy controls (Zhou et al., 2018). In addition, functional ncRNAs are always enriched in exosomes. These features will provide higher sensitivity for exosomal ncRNAs detection in clinical samples, compared to free ncRNAs in body fluids; 3) Stability: Exosomes can protect the contents from enzymes digesting during circulation. ncRNA levels in exosomes will not change significantly after exposure to a variety of stress conditions; 4) Accessibility: Most of the functional exosomal ncRNAs are distributed in blood and can be obtained in a non-invasive manner. Thus, the emerging field of exosomal ncRNAs as biomarkers in leukemia is worth our time and effort.

Despite the strong potential of exosomal ncRNAs as novel therapeutic targets and biomarkers, there are still lots of challenges in developing exosomal ncRNAs for clinical applications. From an investigative perspective, the functions of ncRNAs identified in current studies are usually based on the results obtained from exogenous overexpression of ncRNAs *in vitro* (such as transfecting miRNA mimic) or ncRNAs knockdown in overexpressed cell lines. However, it is hardly known whether the body produces a comparable amount of ncRNAs and promotes the same functions. Similarly, most reports mechanistically analyzing the role of exosomal ncRNAs are conducted in cell line systems or co-culture systems *in vitro* in which the purified exosomes preparations are incubated with intended recipient cells. It is not clear whether the amount of exosome or exosomal ncRNAs applied is within a physiological range. Exogenous supplementation of native and synthetic exosomes may exaggerate the function of exosomal ncRNAs (Sempere et al., 2017). Nevertheless, the critical role of exosomal ncRNAs in tumor development is beyond doubt.

As exosomal ncRNAs are recognized as vital sources of biomarkers for clinical use, it is important to identify the dysregulated ncRNAs in circulating exosomes. Next-generation sequencing (NGS) is a powerful and useful approach for comprehensive transcriptomic profiling and has become an increasingly popular application to analyze ncRNAs in exosomes (Buschmann et al., 2018). However, the circulating exosomes are derived from a variety of cell populations. Their heterogeneous origin will limit the accurate detection of disease-specific exosomes in peripheral blood samples. A vast number of exosomes released from other cell types will dilute the exosome population derived from tumor cells, which reduces the proportion of disease-associated ncRNAs in the sequencing libraries (Huang et al., 2013). Besides, there is no standardized methodology for exosome isolation. Although ultra-centrifugation (UC) is the commonly used method for exosome isolation, other approaches will gain preference when the sample volume is limited, which include filtration, precipitation, sedimentation to size-exclusion chromatography (SEC) and immunocapture (Buschmann et al., 2018). The details in exosome isolation protocols vary among studies and the purity is also difficult to maintain within a certain range, these factors may affect the accuracy and repeatability of the results of NGS or other exosome research (Buschmann et al., 2018). Additionally, compared to cell and tissue samples, the amount of total RNA extracted from exosomes will be far less, therefore a more sensitive exosomal RNA extraction kit is required to ensure the measurement accuracy. The kits from different brands with different protocols will affect the quality of secreted RNA, which may also influence the experiment consequences to some extent. Developing the standard and appropriate exosome isolation and detection methods is a critical step in all areas of exosome research.

From a therapeutic perspective, certain methods of exosome isolation may not be easily scalable to the higher throughput of a hospital laboratory. Although UC is a commonly used method of exosome isolation with high purity and yield, it may require a large volume of blood. The extraction efficiency is also limited by the number of wells in the ultracentrifuge machine, usually six, and the extremely long time the process costs (usually up to 18 h). There are various commercial exosome isolation kits based on filtration, precipitation, SEC and immunocapture which are time-saving and need less sample volume, but the difference of protocols will affect the accuracy and repeatability of detection results (Buschmann et al., 2018). There is a need to standardize methodology and develop technologies that make the isolation and detection of exosomes feasible and scalable to clinical use in hospitals (Desmond et al., 2019). In addition, the exosomes collected from serum or plasma may be contaminated by EVs released by irrelevant cells. How to detect and enrich exosomes secreted by specific tumor cells from complex environments, like heterogeneous cancer tissue or circulation, is also an obstacle for their clinical application. The specific proteins expressed on the membrane of exosomes reflect its origin to some extent. Antibodies designed to target the cell type-specific surface proteins on exosomes may be used to identify and isolate exosomes originating from specific tissues for more accurate clinical detection (Sempere et al., 2017). It has been reported that the epithelial cell adhesion molecule (EpCAM) could be used as a marker to isolate epithelial-derived EVs from the blood sample of colorectal cancer patients by immunoaffinity-capture (Ostenfeld et al., 2016). However, Rupp et al. (2011) demonstrated that EpCAM could be cleaved from exosomes *via* serum metalloproteinases, which may hamper the tumor exosome enrichment by immune-affinity isolation in breast cancer. Further investigations are required to find and develop specific and stable markers for exosome enrichment. Moreover, though the stability of exosome contents makes it possible to apply exosomes in gene therapy, research is still needed to ensure that exosomes containing therapeutic ncRNAs are able to safely escape from the immune system and accurately identify cancerous tissues.

## Conclusion

Understanding the pathogenesis of leukemia and finding specific therapeutic targets and biomarkers is of great importance to improve the clinical efficacy of leukemia patients. Exosomal non-coding RNAs can mediate the communication between cells and the tumor microenvironment by exosome transmission, which is involved in regulating cancerous transformation of mesenchymal cells, fibroblasts, endothelial cells, and advancing tumor cell proliferation, angiogenesis, metastasis, and drug resistance. In addition, exosomal non-coding RNA has excellent characteristics such as tissue or cell specificity, circulation stability, making it a novel biomarker for leukemia identification, treatment monitoring, and efficacy prediction.

Despite the strong potential of exosomal ncRNAs as future therapeutic targets and biomarkers, there are still many challenges that need further research. First, standardization of exosome isolation and purification methodology may increase the accuracy and reliability of functional investigations and advance the clinical application of exosomal ncRNAs. Second, the biological functions of exosomal ncRNAs should be further confirmed *in vivo* and patient samples. The clinical relevance of exosomal ncRNAs in leukemia deserves more extensive exploration. Third, understanding the mechanisms by which donor cells manage to exquisitely load and sort specific ncRNAs cargo into exosomes and how these functional exosomes accurately identify distinct recipient cells without being attacked by the immune system will contribute to the clinical utility of exosomal ncRNAs.

Currently, the understanding of the regulatory function, molecular mechanisms, and clinical relevance of exosomal ncRNAs in leukemia is still limited, especially for circRNA and lncRNA. Further studies on exosomal ncRNAs in leukemia will not only profound impacts on clarifying the pathological process of leukemia but also lay the foundation for novel treatment methods that employ exosomal ncRNAs as biomarkers and therapeutic targets.
